# Optimizing Sensor Ontology Alignment through Compact co-Firefly Algorithm

**DOI:** 10.3390/s20072056

**Published:** 2020-04-06

**Authors:** Xingsi Xue, Junfeng Chen

**Affiliations:** 1 Fujian Key Lab for Automotive Electronics and Electric Drive, Fujian University of Technology, Fuzhou 350118, China; 2 Guangxi Key Laboratory of Automatic Detecting Technology and Instruments (Guilin University of Electronic Technology), Guilin 541004, China; 3 Intelligent Information Processing Research Center, Fujian University of Technology, Fuzhou 350118, China; 4 Fujian Provincial Key Laboratory of Big Data Mining and Applications, Fuzhou 350118, China; 5 College of Information Science and Engineering, Fujian University of Technology, Fuzhou 350118, China; 6 College of IOT Engineering, Hohai University, Changzhou 213022, China; chen-1997@163.com

**Keywords:** sensor ontology, Compact co-Firefly Algorithm, ontology matching

## Abstract

Semantic Sensor Web (SSW) links the semantic web technique with the sensor network, which utilizes sensor ontology to describe sensor information. Annotating sensor data with different sensor ontologies can be of help to implement different sensor systems’ inter-operability, which requires that the sensor ontologies themselves are inter-operable. Therefore, it is necessary to match the sensor ontologies by establishing the meaningful links between semantically related sensor information. Since the Swarm Intelligent Algorithm (SIA) represents a good methodology for addressing the ontology matching problem, we investigate a popular SIA, that is, the Firefly Algorithm (FA), to optimize the ontology alignment. To save the memory consumption and better trade off the algorithm’s exploitation and exploration, in this work, we propose a general-purpose ontology matching technique based on Compact co-Firefly Algorithm (CcFA), which combines the compact encoding mechanism with the co-Evolutionary mechanism. Our proposal utilizes the Gray code to encode the solutions, two compact operators to respectively implement the exploiting strategy and exploring strategy, and two Probability Vectors (PVs) to represent the swarms that respectively focuses on the exploitation and exploration. Through the communications between two swarms in each generation, CcFA is able to efficiently improve the searching efficiency when addressing the sensor ontology matching problem. The experiment utilizes the Conference track and three pairs of real sensor ontologies to test our proposal’s performance. The statistical results show that CcFA based ontology matching technique can effectively match the sensor ontologies and other general ontologies in the domain of organizing conferences.

## 1. Introduction

In recent years, sensors have been used in a wide range of applications, such as urban traffic planning, flood prediction, health care, satellite imaging for earth and space observation et al. To make different types of sensors collaborate on a common task to detect and identify a multitude of observations, we need to combine the sensor data with the Internet, web services and database technologies, which is the so-called sensor web [1,2]. However, since the acquired sensor data on the sensor web lacks of semantic information and may be heterogeneous in the syntax, schema and semantic level and so forth, it is difficult to share and integrate them and implement the communications among diverse sensor applications [3]. To address this problem, Semantic Web (SW) techniques were introduced into the sensor network to annotate the sensor data with spatial, temporal, and thematic semantic meta-data. The combination of the semantic web technology and sensor network leads to the birth of Semantic Sensor Web (SSW) [4], which dedicates to provide semantic meaning for sensor observations so as to enable the inter-operability and advanced analysis for situation awareness and other advanced applications from heterogeneous sensors. In association with semantic annotation, sensor ontologies play an important role in SSW for constructing the shared conceptual model, enhancing the sensor data semantics, and realizing the interaction of spatial, temporal, and thematic sensor data. Annotating sensor data with different sensor ontologies can be of help to implement different sensor systems’ inter-operability, which requires that inter-operable sensor ontologies. To this end, we need to establish meaningful links between semantically related sensor information, which is the so-called sensor ontology matching. Since the sensor ontology can be described through its architecture graph (the nodes represent the concepts and instances, and the edges the relationships between them), the sensor ontology matching process corresponds to the determination of the largest isomorphic sub graph between two architecture graphs of two ontologies to be aligned [5]. Therefore, modeling two sensor ontologies under alignment is a complex and time-consuming task, particularly when the scale of their entities is large. For this reason, approximate methods, such as Swarm Intelligent Algorithm (SIA), represents a suitable methodology for determining the high-quality alignments [6].

With the development of SSW, the scale of sensor ontologies increase significantly, which makes the traditional SIA-based approaches unable to efficiently determine the high-quality alignment due to the huge memory consumption, long runtime and premature convergence. To reduce the memory consumption and runtime, we first investigate a new category of efficient optimization algorithm, that is, Compact Optimization Algorithm (COA), which makes use of the probability representation to approximately present the population. Since COA only needs to restore a Probability Vector (PV), it significantly saves the hardware resources to execute the evolving process. The first COA is the Compact Genetic Algorithm (CGA) [7], and later, Baraglia et al. [8] improve its performance by introducing a local search strategy. Ahn et al. [9] use two elite strategies to enhance CGA’s performance. Neri et al. [10] propose a noise robust version to deal with the situation with noise context. They also propose a Compact Particle Swarm Optimization (CPSO) [11] to approximate the swarm’s behaviour. Mininno et al. propose a Compact Differential Evolution algorithm (CDE) [12], whose survivor selection scheme can be straightforwardly encoded, which can efficiently perform an optimization process with a limited memory requirement.

In addition, to overcome the algorithm’s premature convergence, we also investigate another kind of optimization algorithm, that is, co-Evolutionary Algorithm (cEA), which makes multiple sub-swarms evolves independently and exchanges the information of each sub-swarms at particular time to improve the searching efficiency in the large search space. Tan et al. [13] first decomposes the problem’s vector dimensions, and then tries to make each sub-swarm addresses one sub-problem. Mu et al. [14] use the multiple elite strategies to execute the evolving process, and they assign the centered sub-swarm with higher priority. Zhou et al. propose a Parallel cEA (PcEA), which divides the population into three sub-swarms and they evolve with the same evolving strategy. To improve the algorithm’s search ability, Liu et al. [15] propose two elite strategies, that is, a better elite strategy and a worse elite strategy. They utilize two sub-swarms with different evolving strategies to respectively update two kinds of elites. EI-Abd [16] propose a Cooperative Co-evolutionary Brain Storm Optimization algorithm (CCBSO), which is based on the explicit space decomposition approach. In this work, we combines the COA with cEA to have their complementary advantages, and being inspired by the success of Firefly Algorithm (FA) in many domains [17,18], we further propose a general-purpose ontology matching technique based on Compact co-Firefly Algorithm (CcFA) to efficiently optimize the ontology alignments.

The rest of the paper is organized as follows: Section 3 presents the basic concepts on sensor ontology matching; Section 4 shows the CcFA-based sensor ontology matching technique in details; Section 5 presents the greedy strategy to filtering the final alignment; Section 6 shows the statistical experiment; and finally, Section 7 draws the conclusion and presents the future work.

## 2. Swarm Intelligence Algorithm Based Ontology Alignment

Many Machine Learning (ML) techniques have been applied to match ontologies to determine high-quality alignment, such as Logistic Regression (LR) [19], Neural Network (NN) [20], Word Embedding (WE) [21], Graph Embedding (GE) [22], Support Vector Machine (SVM) [23], Clustering Algorithm [24], Decision Tree (DT) [25] and so forth. Researchers also try to improve the matching efficiency through the high performance computing techniques such as Parallel Computing (PC) [26] and Cloud Computing (CC) [27]. Due to the complexity of the ontology matching process, recently, SIA-based technique has become an efficient approach for determining high-quality ontology alignment.

The first generation of SI-based matchers aimed at solving the ontology meta-matching problem, that is, how to determine the optimal parameters to aggregate different matchers and optimize the quality of obtained ontology alignment. Genetics for Ontology ALignments (GOAL) [28] was the first SI-based ontology meta-matcher, which used Evolutionary Algorithm (EA) to optimize the aggregating weight set of different ontology matchers. Ginsca et al. [29] proposed to use EA to optimize the all the parameters in the whole meta-matching process, which included the aggregating weight set and a threshold for filtering the final alignment. Xue et al. [30] introduced a new metric to measure the ontology alignment’s quality, which did not require the utilization of golden standard alignment, and formally defined ontology meta-matching problem. Their approach was able to match multiple ontology pairs at a time and overcame three drawbacks of the EA-based meta-matchers. More recently, He et al. [31] used Artificial Bee Colony (ABC) algorithm to address the ontology meta-matching problem, whose results were better than the EA-based matchers. Recently, Xue et al. [32] propose a Multi-objective CFA (MCFA) to optimize the ontology alignments. Their proposal borrows the idea of MOEA/D [33] by first decomposing the original problem into three sub-problems, and then use three PVs to respectively address three sub-problems. Later on, Xue [34] further construct a single objective model for the biomedical ontology matching problem, and proposes a Compact Firefly Algorithm (CFA) to address it. CFA utilizes the compact encoding mechanism to represent the swarm of fireflies with a probabilistic model, instead of storing each one, but it may result in premature convergence. To overcome this drawback, in this work, CcFA combines the compact encoding mechanism with the co-Evolutionary mechanism to further enhance the algorithm’s performance.

## 3. Sensor Ontology Matching Problem

An ontology can be defined as a 3-tuples (C,DP,OP), where *C* is the set of classes, that is, the set of concepts that populate the domain of interest; DP is the set of data properties, that is, the set of features describing the classes; OP is a set of object properties, that is, the set of relations existing between the concepts. In particular, concept, datatype property and object property are called ontology entities. Sensor ontology formally defines the shared concepts and their relationships in the sensor domain. However, since the sensor ontologies is developed and maintained by different organizations with various requirements, they may define the same observation with different ways, which yields the sensor ontology heterogeneity problem. It is necessary to find the identical sensor entities to bridge the semantic gap between two ontologies, which is the so-called sensor ontology matching. The obtained sensor entity mapping set is called sensor ontology alignment.

It is obvious that how to calculate two sensor entity’s similarity value is the prerequisite technique for matching sensor ontologies [35]. Since none of the similarity measure can ensure their effectiveness in any context, usually, a sensor ontology system aggregates several similarity measures to improve the result’s confidence. In this work, we combine three broad categories of similarity measures to measure two sensor entities’ similarity, that is, structure-based, linguistic-based and syntax-based similarity measures. To be specific, we construct a context profile for each sensor entity by collecting the information from its direct ascendant and descendant entities. Then, the similarity of two entities $e_{1}$ and $e_{2}$ is calculated as follows:

(1) $$sim\left(e_{1},e_{2}\right)=\frac{2\times |p_{1}⋂p_{2}|}{|p_{1}\left|+\right|p_{2}|},$$

where $p_{1}$ and $p_{2}$ are respectively $e_{1}$ and $e_{2}$’s profiles. With respect to the similarity measure on two elements $w_{1}$ and $w_{2}$ in two profiles, we aggregate the Wordnet [36] based distance, a linguistic-based similarity measure, and the N-gram distance [37], a syntax-based similarity measure, whose equation is as follows:

(2) $$sim\left(w_{1},w_{2}\right)=\left\{\begin{array}{cc} 1, & \mathrm{two words are synonymous}\\ N-gram(w_{1},w_{2}), & \mathrm{otherwise} \end{array}.\right.$$

Since the alignment with more correspondences and higher mean similarity value should have better quality, the quality of a sensor ontology alignment is calculated as follows:

(3) $$f\left(A\right)=2\times \frac{\frac{\left|A\right|}{max\{|O_{1}|,|O_{2}|\}}\times \frac{\sum_{i=1}^{\left|A\right|}sim_{i}}{\left|A\right|}}{\frac{\left|A\right|}{max\{|O_{1}|,|O_{2}|\}}+\frac{\sum_{i=1}^{\left|A\right|}sim_{i}}{\left|A\right|}},$$

where $|O_{1}|$, $|O_{2}|$ are respectively the entity number of two sensor ontology $O_{1}$ and $O_{2}$, |A| is the mapping number of the alignment *A*, and $sim_{i}$ is the *i*-th entity mapping’s similarity. Further, the sensor ontology matching problem is defined as follows:

(4) $$\left\{\begin{array}{c} max\;f(X)\\ s.t.\;X={\left(x_{1},x_{2},\ldots ,x_{|O_{1}|}\right)}^{T}\\ \;\;x_{i}=1,2,\cdots ,\left|O_{2}\right| \end{array},\right.$$

where $x_{i}$ is the *i*th entity mapping between *i*th entity in $O_{1}$ and $x_{i}$th entity in $O_{2}$.

## 4. Compact co-Firefly Algorithm

FA is inspired by the social behaviour of fireflies, where fireflies produce short and rhythmic flashes for communication. The intuition behind FA is that fireflies tend to fly to the brighter locations, which is a solution with better objective function’s value [18]. In this work, we propose a Compact co-Firefly Algorithm, that is, CcFA, which utilizes the compact encoding mechanism and the co-Evolutionary mechanism to improve the solution’s quality. CcFA works with two sub-swarms, that is, the better swarm with a better elite solution and a worse swarm with a worse elite solution, which respectively apply different evolving strategy. In particular, the better swarm mainly focus on the exploitation which can increase the convergence speed, while the worse swarm emphasizes on the exploration which is able to enhance the solution’s diversity. In each generation, the one with a better elite solution will become the better swarm, and in this way, two swarms can better trade off the algorithm’s exploitation and exploration. To improve the searching performance, we utilize two Probability Vectors (PVs) to represent two sub-swarms, that is, $PV_{better}$ and $PV_{worse}$.

### 4.1. Compact Encoding Mechanism

**Figure 1:**
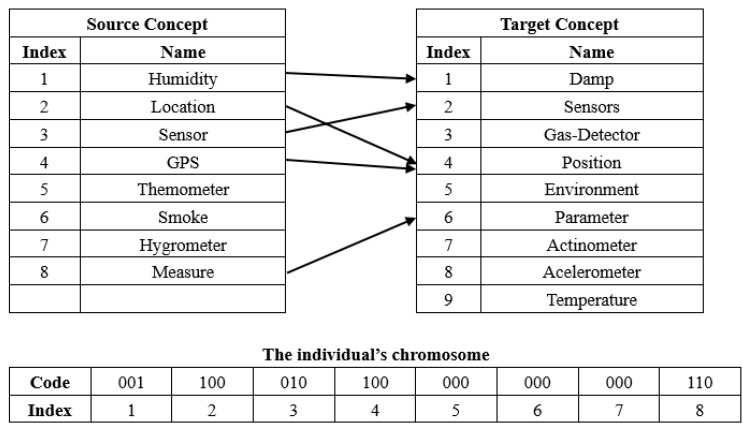
An example of compact encoding mechanism.

An alignment consists of several entity mappings, and each entity mapping can be described by two entities’ indices. On this basis, in this work, we empirically choose the Gray code to encode an alignment, which is a binary encoding mechanism. An example of the compact encoding mechanism is shown in the Figure 1, where the index means the source concept index and the corresponding bit values are the target concept index that is encoded through Gray code, for example, the source concept “Measure” with index 8 is mapped to target concept “Parameter” with index 6 whose Gray code is 110. In particular, Gray code 000 means a source concept is not mapped to any target concept. We need to point out that, given the the scale of target ontology *n*, the length of code is equal to $\left\lceil log_{2}n\right\rceil$, and Figure 1 only shows a simple example of encoding with Gray code.

**Figure 2:**
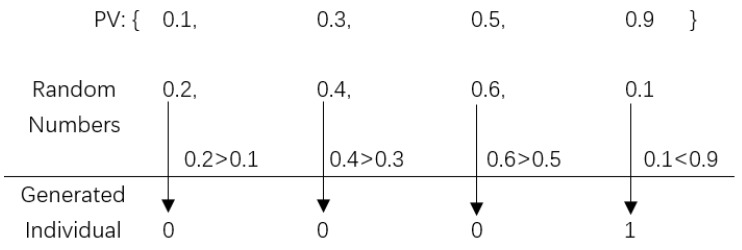
An example of generating an individual through PV.

A PV’s dimension number is equal to the length of an individual, and each dimension represents the probability of being one corresponding to each individual bit’s value. Since PV stores each individual bit’s corresponding probability of being one, each bit value of a newly generated individual can be determined by comparing a random number with its corresponding probability in PV. Figure 2 shows an example of generating a solution through PV. Given a PV ${\left(0.1,0.3,0.5,0.9\right)}^{T}$ where each element presents the probability of being 1 with respect to a solution’s gene bit, generate four random numbers in [0,1], for example, 0.2, 0.4, 0.6 and 0.1, and we can determine a new solution by comparing them with PV’s elements accordingly. To be specific, since 0.2 > 0.1, 0.4 > 0.3, 0.6 > 0.5 and 0.1 < 0.9, the newly generated solution is 0001. In addition, PV is updated in each generation to make the new individuals generated in the next are closer to the elite. With respect to the word “closer”, it means the newly generated individual is more likely to be the elite solution. For example, given two PVs $PV_{1}={\left(0.1,0.1,0.1,0.1\right)}^{T}$ and $PV_{2}={\left(0.9,0.9,0.9,0.9\right)}^{T}$, it is obvious that a new individual generated by $PV_{2}$ is more likely to be 1111 than that generated by $PV_{1}$. Therefore, if the elite 1111 is the global optimal solution, we need to update $PV_{1}$ by moving it towards $PV_{2}$. In particular, if the bit value of the elite solution is 1 (or 0), PV’s first element will be increased (decreased) by a step, which can make the newly generated solution more closer to the elite solution. Given a PV ${\left(0.1,0.3,0.5,0.9\right)}^{T}$, an elite solution 1110 and step=0.1, since the first bit value of the elite solution is 1, accordingly, we update the first element of PV by step, that is 0.2, which makes the first bit value of newly generated individual is more likely to be 1 (the same with elite solution’s first bit value). Therefore, after updating all elements of PV, the newly generated individuals would be closer to the elite solution in terms of each bit value. When all elements of PV are closed to 1 or 0, the newly generated individuals will be same and the algorithm converges. For more about the theory analysis on the compact encoding mechanism, please see also the work of Harik et al. [7].

### 4.2. Movement Operator

In the classic FA, a firefly $ind_{i}$’s position is updated by moving it to a more attractive firefly $ind_{j}$ by the α-step and β-step, which are respectively given as follows:

(5) $$\alpha -step\left(ind_{i}\right)=ind_{i}+\alpha \left(rand\left(\right)-1/2\right).$$

(6) $$\beta -step\left(ind_{i}\right)=\frac{\beta_{0}}{1+\gamma r_{ij}^{2}}r_{ij},$$

where α is a randomisation function, $r_{ij}$ is two fireflies’ distance, $\beta_{0}$ is the attractiveness when $r_{ij}=0$, γ is the light absorption parameter.

#### 4.2.1. Exploitation Strategy

FA’s exploitation searches for the better individual in the vicinity of a solution, which is implemented through α-step. In this work, α-step can be implemented through a local search process on $ind_{i}$. Given a firefly ind, we first generate *C* new individuals through PV; then, utilize the binary crossover operator on each of the new individual and ind to obtain ind’s neighborhood, finally, we select the elite from its neighborhood. The pseudo-code of α-step algorithm is shown in Algorithm 1.
**Algorithm 1**α-step Algorithm **for** int i=0; i<C, *i*++ **do**  generate a new solution $ind_{new}$ through Probability Vector (PV);  $ind_{i}=ind.copy\left(\right)$;  int index=round(rand(0,C));  **while** rand(0,1)<0.6 **do**   $ind_{i,index}=ind_{new,index}$;   index++;   **if** index==C **then**
    index=0;   **end if**  **end while** **end for** return the best individual in $\left\{ind_{1},ind_{2},\cdots ,ind_{C}\right\}$

#### 4.2.2. Exploration Strategy

FA’s exploration aims at keeping the the population’s diversity, which is implemented through β-step. In this work, we utilize the edit distance to measure two fireflies $ind_{i}$ and $ind_{j}$’s distance:

(7) $$edit\left(ind_{i},ind_{j}\right)=\sum_{k=1}^{|ind_{i}|}|ind_{i,k}-ind_{j,k}|,$$

where $|ind_{i}|$ is the number of $ind_{i}$’s bits, $ind_{i,k}$ and $ind_{j,k}$ are respectively the *k*th bit of $ind_{i}$ and $ind_{j}$. Next, a new individual $ind_{i}$ can be obtained according to $edit(ind_{r},ind_{s})$ and $\beta_{0}/\left(1+\gamma r_{ij}^{2}\right)$. The pseudo-code of β-step algorithm is shown in Algorithm 2.

When the edit distance between two PVs is too close, all $PV_{worse}$’s elements will be initialized. In particular, the edit distance between two PVs are defined as follows:

(8) $$edit\left(PV_{better},PV_{worse}\right)=\frac{\sum_{i=1}^{\left|PV\right|}(|PV_{better}^{i}-PV_{worse}^{i}\left|\right)}{\left|PV\right|},$$

where |PV| is PV’s dimension number, $PV_{better}^{i}$ and $PV_{worse}^{i}$ are respectively the *i*th dimension of the $PV_{better}$ and $PV_{worse}$.
**Algorithm 2**β-step Algorithm **for** k=0; $k<|ind_{i}|$; *k*++ **do**  **if** $ind_{i}\left[k\right]!=ind_{j}\left[k\right]$ **then**   put *k* into the list index;  **end if** **end for** $totalNum=round(rand\left(0,1\right)\times Hamming\left(ind_{i},ind_{j}\right))$; num=0; n=0; **while** num<totalNum **do**
  **if** $rand\left(0,1\right)<\frac{\beta_{0}}{1+\gamma r_{ij}^{2}}$ **then**
   $ind_{i}\left[index\left[n\right]\right]=\left(ind_{i}\left[index\left[n\right]\right]+1\right)\;mod\;2$;   remove index[n] from index;   num=num+1;  **end if**  n=(n+1)modindex.length(); **end while**

### 4.3. Pseudo-Code of Compact co-Firefly Algorithm

In this work, CcFA uses the following configuration: the maximum generation maxGen=3000, the attractiveness $\beta_{0}=1.0$, the light absorption parameter γ=0.02, the local search’s neighborhood scale C=5, the step length for updating PV step=0.1. These parameters represent a trade off configuration obtained in an empirical way to achieve the highest average alignment quality on all testing cases. CcFA’s pseudo-code is shown in Algorithm 3.

CcFA applies two evolutionary strategies on $PV_{better}$ and $PV_{worse}$, respectively. Through the competition between two elites, $PV_{better}$ and $PV_{worse}$ are updated. By adaptively switching the search strategies, CcFA can better trade off the algorithm’s converging speed and the individuals’ diversity. In particular, when all elements of $PV_{better}$ and $PV_{worse}$ are equal to 1 or 0, CcFA converges, and the algorithm terminates the loop and outputs $elite_{better}$.
**Algorithm 3** Compact co-Firefly Algorithm ** Initialization ** generation=0; Initialize $PV_{better}$ and $PV_{worse}$ by setting all the probabilities inside as 0.5; generate $elite_{better}$ and $elite_{worse}$ through $PV_{better}$ and $PV_{worse}$, respectively; ** Evolving Process ** **while** generation<maxGen **do**
  ** Exploitation **  generate a solution $ind_{new}$ through $PV_{better}$;  $ind_{new}$ = alpha-step($ind_{new}$); // see also Algorithm 1  ** Competition **  $compete(elite_{better},ind_{new})$;  **if** $winner==ind_{new}$ **then**   $elite_{better}=ind_{new}$;  **end if**  ** PV Update **  **for** i=0; $i<PV_{better}.length$; *i*++ **do**   **if** winner[i]==1 **then**    $PV_{better}{\left[i\right]}_{better}=PV_{better}\left[i\right]+step$;   **else**    $PV_{better}\left[i\right]=PV_{better}\left[i\right]-step$;   **end if**  **end for**  ** Exploration **  generate a solution $ind_{new}$ through $PV_{worse}$;  $ind_{new}$ = beta-step($ind_{new}$, $ind_{worse}$); // see also Algorithm 2  ** Competition **  $compete(elite_{worse},ind_{new})$;  **if** $winner==ind_{new}$ **then**   swap($elite_{worse},ind_{new})$;  **end if**  ** PV Update **  **for** i=0; $i<PV_{worse}.length$; *i*++ **do**   **if** winner[i]==1 **then**    $PV_{worse}\left[i\right]=PV_{worse}\left[i\right]+step$;   **else**    $PV_{worse}\left[i\right]=PV_{worse}\left[i\right]-step$;   **end if**  **end for**  ** Communication **  $compete(elite_{better},elite_{worse})$;  **if** $winner==elite_{worse}$ **then**   swap($elite_{better},elite_{worse})$;   swap($PV_{better},PV_{worse})$;  **end if**  update $PV_{worse}$; // see also Section 4.2.2  ** Judge whether the algorithm converges **  **if** all elements of $PV_{better}$ and $PV_{worse}$ are equal to 1 or 0 **then**   break;  **end if** **end while** output $elite_{better}$;

## 5. Final Alignment Determination

**Figure 3:**
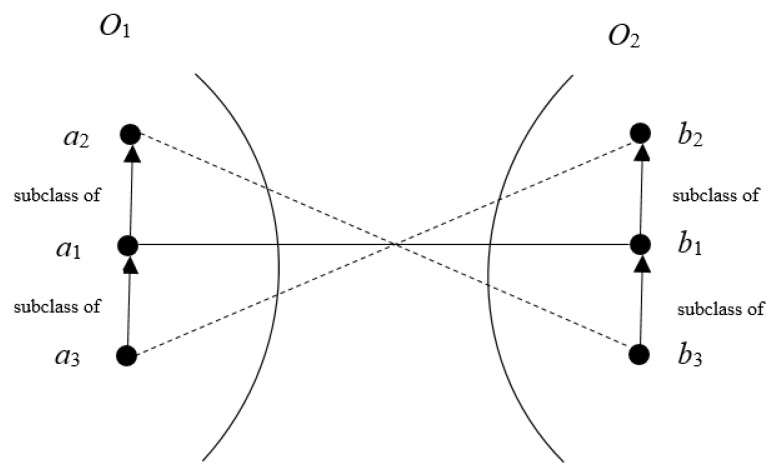
An example of inconsistent mappings.

In this work, we utilize a greedy heuristic to filter the alignment. First, we remove those correspondences with similarity value lower than 0.88 from the obtained alignment to ensure the precision of the final alignment. Here, we utilize the threshold 0.88 by referring to Fernandez et al. [38]. Then, we sort the resting correspondences by descending similarity, and select them one by one into the final alignment as long as they do not conflict with previous selected ones. In this work, we use a logic reasoning approach to judge whether two correspondences conflict with each other. According to Wang [39], an ontology’s entities are often organized by their “is-a” relationships, and a correct alignment should be consistent with that hierarchy. In Figure 3, if the correspondences $\left(a_{1},b_{1}\right)$ has high similarity values, that is, $a_{1}$ matches $b_{1}$, the mapping between $a_{1}$’s super-concepts $a_{2}$ (or sub-concept $a_{3}$) and $b_{1}$’s sub-concept $b_{3}$ (or super-concept $b_{2}$), that is, $\left(a_{2},b_{3}\right)$ and $\left(a_{3},b_{2}\right)$ both conflict with $\left(a_{1},b_{1}\right)$.

## 6. Experiment

### 6.1. Experimental Configuration

In the experiments, the OAEI’s Conference track with ra1 version [40] and three pairs of real sensor ontologies are used to test CcFA’s performance. We compare CcFA with two SIA-based ontology matching techniques, that is, EA-based matcher [41], PSO-based matcher [42] and four state-of-the-art sensor ontology matching systems, that is, SOBOM [43], CODI [44], ASMOV [45] and FuzzyAlign [38], whose code and configuration parameters are available online. SOBOM works based on the syntax and structure based similarity measures, and it can obtain better results when the literal of concept and ontology hierarchy structure is complete. CODI utilize the syntax based similarity measure and the Markov Logic based probabilistic model to produce the alignment. ASMOV determines the alignment through an iterative way, and it also takes into consideration three kinds of similarity measure to calculate the similarity values. FuzzyAlign use a fuzzy rule-based strategy to combine three categories of similarity measure, and it utilizes the EA to determine a threshold for filtering the final alignment.

The obtained alignments are evaluated through the f-measure [46], which works based on the golden standard alignment. EA, PSO and CcFA’s results are the mean values of thirty independent runs. CcFA use the parameters (see also Section 4.3) which can ensure the highest average alignment quality on all exploited testing cases, EA and PSO utilize the configurations according to their own literatures, and all SIA-based techniques’ results are the average of thirty independent runs.

### 6.2. The Results of Statistical Comparison

The conference track requires matching seven ontologies describing the domain of organizing conferences, that is, Cmt (http://msrcmt.research.microsoft.com/cmt), Pcs (http://precisionconference.com), OpenConf (http://www.zakongroup.com/technology/openconf.shtml), Edas (http://edas.info/), Ekaw (http://ekaw.vse.cz), Iasted (http://iasted.com/conferences/2005/cancun/ms.htm) and Sigkdd (http://www.acm.org/sigs/sigkdd/kdd2006). Three pairs of real sensor ontologies are MMI Device ontology vs SSN ontology, CSIRO sensor ontology vs SSN ontology, and MMI Device ontology vs CSIRO sensor ontology. In particular, MMI Device Ontology (http://mmisw.org/ont/mmi/device) is developed by the Marine Metadata Project (MMP) for promoting the exchange, integration and use of marine data; SSN ontology (https://www.w3.org/TR/vocab-ssn) is developed by the World Wide Web Consortium (W3C) for modeling the knowledge in the sensor network domain; and CSIRO sensor ontology (https://www.w3.org/2005/Incubator/ssn/wiki/SensorOntology2009) is developed by M. Compton et al. from CSIRO (Australia) for providing a semantic description of sensors in terms of the sensor grounding and operation specification. All these sensor ontoloies are all widely used and open to achieve. The reasons that we select SSN ontology, CSIRO sensor ontology and MMI Device ontology in the experiment are: (1) they have defined lots of overlapping information with different representations; (2) SSN is one of the most used global reference ontologies, and it provides the alignment with another upper ontology–DOLCE ultra lite (http://ontologydesignpatterns.org/ont/dul/DUL.owl), which can be used as the golden alignment to measure an alignment’s quality, that is, calculate the f-measure value. Table 1 shows the statistical information about the above ontologies.

We utilize the Friedman’s test [47] and Holm’s test [48] to carry out the statistical experiment in terms of the alignments’ quality. In particular, Friedman’s test [47] is used to figure out whether there are any differences among these competitors, and Holm’s test [48] is used to check whether one competitor statistically outperforms others. In Friedman’s test, we need to reject the null-hypothesis that all the competitors are equivalent. Therefore, the computed value $X_{r}^{2}$ must be equal to or greater than the tabled critical chisquare value at the specified level of significance α. Here, we choose α=0.05, and we need to consider the critical value for 6 degrees of freedom since we are comparing 7 matchers, that is, $X_{0.05}^{2}=12.592$.

In Table 2, each value represents the f-measure, and the number in round parentheses is the corresponding computed rank. The computed $X_{r}^{2}=119.73$, which is greater than 12.592, and therefore, the null hypothesis is rejected. Then, the Holm’s test is further carried out. As shown in Table 2, since our approach ranks with the lowest value, it is set as a control matcher that will be compared with others.

**Figure 4:**
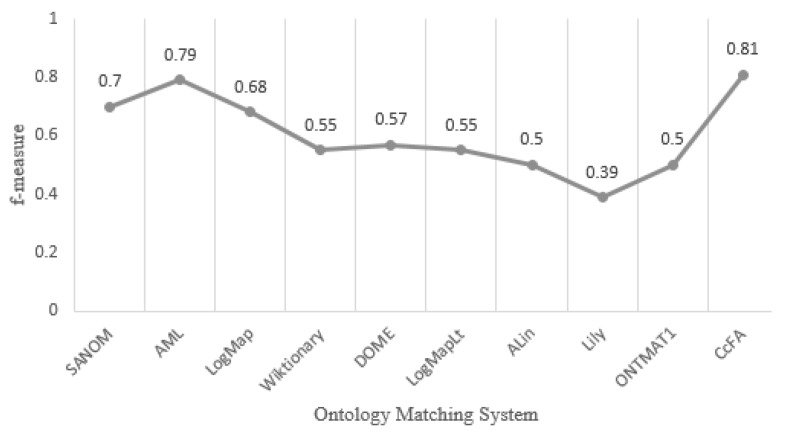
Comparison with state-of-the-art ontology matching systems on the Conference track.

In Holm’s test, z−value is the testing statistic for comparing the *i*th and *j*th matchers, which is used for finding the *p*-value (the corresponding probability from the table of the normal distribution). *p*-value is then compared with α=0.05, which is an appropriate level of significance. According to Table 3, we can state that CcFA statistically outperforms other competitors on f-measure at 5% significance level. In Figure 4, we compare CcFA with OAEI’s participants (http://oaei.ontologymatching.org/2019/results/conference/index.html) in terms of average f-measure.

As can be seen from the tables and figure, the f-measure values obtained by CcFA outperform all the other competitors, which shows that CcFA can effectively optimize the ontology alignments. In particular, the quality of alignment of CcFA is better than EA and PSO, which shows that CcFA’s compact encoding mechanism and compact operators can better trade off the algorithm’s exploration and exploitation. Since none of the similarity measures can effectively distinguish all the heterogeneous concepts in any situations, it is necessary to aggregate several similarity measures to improve the result’s precision. We utilize a hybrid similarity measure which combines three kinds of similarity measures to calculate the entity similarity value, and therefore CcFA’s results are significantly higher than other systems that only take into consideration one or two categories of similarity measure, such as SOBOM, CODI, DOME, Lily, ALin, LogMap family and Wiktionary. However, FuzzyAlign, ASMOV, AML, SANOM and ONTMAT1 apply too many similarity measures that lead to the conflicting results, which decreases the recall value. Thus, how many similarity measures should be selected and combined to ensure the quality of the alignment will be one of our future work.

Currently, there is a new trend for developing lightweight sensor ontologies, such as the Sensor, Observation, Sample, and Actuator (SOSA) ontology [49], which only provides the specification for the kernel SSN’s entities that involves in the acts of observation, actuation and sampling, and IoT-Lite [50]. CcFA can also represent an efficient approach for matching these lightweight sensor ontologies since they own less entities and the search space is relatively smaller. However, since the entities’ semantic relationships in the lightweight ontologies could be more complicated and richer, lightweight sensor ontology alignment is more complex, that is, one source ontology entity is mapping with more than one target ontology entity, and the relationships could be equivalence or subsumption. To address this complex matching problem [51], a feasible method is to introduce various mapping patterns [52] into CcFA to detect the complex correspondences, which is one of our future work.

## 7. Conclusions and Future Work

In order to support the information integration of various sensor ontologies, in this paper, a general-purpose ontology matching technique based on CcFA is proposed. Our proposal makes use of compact α-step and β-step to implement the discrete exploitation and exploration, and trade off them during the evolving process through two PVs. The experiment shows the effectiveness of this combination of compact encoding mechanism and co-Evolutionary mechanism, and our approach statistically outperforms other competitors on alignment’s quality at 5% significance level when matching sensor ontologies and other ontologies in the domain of organizing conferences.

In the future, we will further study the technique that can adaptively select and combine various similarity measures according to different heterogeneity situation. Moreover, we will improve CcFA based approach to match the large-scale sensor ontologies, which is a challenge in the ontology matching domain. Another challenge in ontology matching domain is the problem of Instance Coreference Resolution (ICR) [53] in the sensor network domain, which requires matching large-scale sensor instances in the Linked Open Data cloud (LOD). Currently, there is no SIA-based technique that could effectively solve ICR, and we are also interested in addressing this challenge with CcFA. In this work, we mainly aim at matching sensor ontologies, as well as other general ontologies, such as those in the domain of organizing conferences. With respect to the specific ontologies like biomedical ontology and geographical ontology, directly applied our proposal to match them could yield low precision and recall because these tasks require specific background knowledge base and complex forms of alignment. Therefore, we would like to extend the CcFA-based matching technique to address these matching tasks in the specific domains.

## Figures and Tables

**Table 1:** A brief description on the sensor ontologies.

Name	Class Scale	Datatype Property Scale	Object Property Scale
Cmt	36	10	49
Pcs	23	14	24
OpenConf	62	21	24
Edas	104	20	30
Ekaw	77	0	33
Iasted	140	3	38
Sigkdd	49	11	17
MMI Device ontology	55	43	28
SSN ontology	19	2	36
CSIRO sensor ontology	75	11	61

**Table 2:** Friedman’s test on the alignment’s quality.

Matching Task	SOBOM	CODI	ASMOV	FuzzyAlign	EA	PSO	CcFA
**OAEI’s Conference Track**							
Cmt-Pcs	0.50(7)	0.75(5)	0.59(6)	0.87(2)	0.82(4)	0.85(3)	**0.91(1)**
Cmt-OpenConf	0.21(7)	0.38(5)	0.28(6)	0.45(3)	0.40(4)	**0.48(1.5)**	**0.48(1.5)**
Cmt-Edas	0.48(6)	0.75(5)	0.42(7)	0.86(1.5)	0.78(3.5)	0.78(3.5)	**0.86(1.5)**
Cmt-Ekaw	0.52(7)	0.75(3)	0.59(6)	0.88(2)	0.66(4)	0.64(5)	**0.92(1)**
Cmt-Iasted	0.54(6)	0.78(5)	0.50(7)	0.87(2.5)	0.82(4)	0.87(2.5)	**0.90(1)**
Cmt-Sigkdd	0.14(7)	0.68(2)	0.34(6)	0.61(4)	0.60(5)	0.64(3)	**0.72(1)**
Pcs-OpenConf	0.40(7)	0.75(4)	0.51(6)	0.86(2)	0.74(5)	0.80(3)	**0.89(1)**
Pcs-Edas	0.44(7)	0.75(5)	0.50(6)	**0.88(1.5)**	0.83(4)	0.86(3)	**0.88(1.5)**
Pcs-Ekaw	0.38(6)	0.70(5)	0.32(7)	0.87(2)	0.83(4)	0.86(3)	**0.90(1)**
Pcs-Iasted	0.54(6)	0.73(5)	0.50(7)	0.89(2)	0.84(3)	0.82(4)	**0.93(1)**
Pcs-Sigkdd	0.40(7)	0.70(5)	0.59(6)	0.80(2)	0.77(3)	0.75(4)	**0.85(1)**
OpenConf-Edas	0.14(7)	0.36(5)	0.27(6)	0.59(2)	0.50(3)	0.56(4)	**0.67(1)**
OpenConf-Ekaw	0.25(7)	0.41(4.5)	0.28(6)	0.52(2)	0.44(3)	0.41(4.5)	**0.54(1)**
OpenConf-Iasted	0.20(7)	0.70(5)	0.31(6)	0.71(4)	**0.79(1.5)**	0.75(3)	**0.79(1.5)**
OpenConf-Sigkdd	0.35(7)	0.61(5)	0.48(6)	0.82(2.5)	0.82(2.5)	0.79(4)	**0.85(1)**
Edas-Ekaw	0.10(7)	0.25(6)	0.39(4)	0.44(2)	0.34(5)	0.41(3)	**0.54(1)**
Edas-Iasted	0.32(6)	0.72(3)	0.20(7)	0.84(2)	0.62(5)	0.70(4)	**0.88(1)**
Edas-Sigkdd	0.25(7)	0.58(5)	0.33(6)	0.66(3)	0.69(2)	0.65(4)	**0.73(1)**
Ekaw-Iasted	0.30(7)	0.64(5)	0.37(6)	0.87(2)	0.81(4)	0.82(3)	**0.94(1)**
ekaw-Sigkdd	0.22(7)	0.78(3)	0.25(6)	**0.74(1.5)**	0.70(4)	0.68(5)	**0.74(1.5)**
Iasted-Sigkdd	0.17(7)	0.72(5)	0.20(6)	0.77(2)	0.74(3.5)	0.74(3.5)	**0.83(1)**
**Three Pairs of Real Sensor Ontologies**							
MMI Device-SSN	0.77(7)	0.80(5)	0.73(6)	0.88(2)	0.82(4)	0.85(3)	**0.92(1)**
CSIRO-SSN	0.78(5.5)	0.79(4)	0.75(6)	0.88(2)	0.78(5.5)	0.87(3)	**0.94(1)**
MMI Device-CSIRO	0.72(7)	0.78(4)	0.75(5)	0.87(2)	0.74(6)	0.82(3)	**0.90(1)**
Average	0.38 (6.72)	0.66 (4.52)	0.43 (6.08)	0.76 (2.22)	0.70 (3.85)	0.72 (3.43)	**0.89 (1.10)**

**Table 3:** Holm’s test on the alignment’s quality.

*i*	Approach	z-Value	Unadjusted p-Value	$\frac{\alpha }{k-i},\alpha =0.05$
8	FuzzyAlign	2.22	0.030	0.050
7	EA	3.43	1.91 $\times 10^{-4}$	0.025
5	PSO	3.85	1.08 $\times 10^{-5}$	0.012
3	CODI	4.52	4.25 $\times 10^{-8}$	0.008
2	ASMOV	6.08	1.55 $\times 10^{-15}$	0.007
1	SOBOM	4.45	4.24 $\times 10^{-17}$	0.006
